# Continuity and discontinuity in infant and maternal behavior from 3 to 9 months according to prematurity status

**DOI:** 10.3389/fnbeh.2024.1303393

**Published:** 2024-02-21

**Authors:** Marina Fuertes, Joana L. Gonçalves

**Affiliations:** ^1^Escola Superior de Educação de Lisboa, Instituto Politécnico de Lisboa, Lisboa, Portugal; ^2^Center for Psychology at University of Porto, Faculty of Psychology and Education Sciences, University of Porto, Porto, Portugal; ^3^Center for Research in Psychology for Positive Development, Lusíada University, Porto, Portugal; ^4^Department of Social and Behavioral Sciences, University of Maia, Maia, Portugal

**Keywords:** early interactions, continuity and discontinuity, infant and maternal interactive behavior, prematurity, infancy

## Introduction

Maternal sensitivity is the stronger predictor of attachment (Madigan et al., 2023) and is associated with infant development (Bigelow et al., 2004; Deneault et al., 2023). Also, it is associated with health indicators and medical practices (Fuertes et al., 2020a; Dionisio et al., 2023). Mothers' sensitive behavior is characterized by affectionate, appropriate, contingent, and consistent responsiveness to infants' social cues and needs (Crittenden, 1999). Considering this definition, maternal sensitivity is interpreted in the context of a mother-infant interchange, in which infants' needs, responses, and dyadic interchanges are of utter importance.

Social (e.g., maternal education) and infant (e.g., birthweight) factors affect maternal sensitivity and infant cooperative behavior (Neuhauser, 2018). In prior research from our lab (Fuertes et al., 2024), we found differences in maternal and infant behavior according to infant prematurity status using the samples of these studies. Maternal sensitivity and infant cooperativity were high in dyads with infants born FT (full-term), moderate in dyads with infants in the MLPT (moderate-to-late preterm) group, and low in dyads with infants born VEPT (very-to-extreme preterm). Moreover, infants born VEPT were more difficult and less passive than the other two groups, and their mothers were more unresponsive. However, little is known about if and how maternal sensitivity and infant cooperativity change (discontinuity) from 3 to 9 months in those groups.

Differences in maternal and infant behavior can be explained by the accumulation of experiences resulting from mother-infant interactions. This allows infants to establish an expectation of other responses, dyadic reciprocity, and contingency. Both infants and adults contribute to the development of relationships, which require individual and dyadic adaptation, promoting mutual shared intersubjectivity (Tronick, 2007).

Infants' developmental processes throughout the 1st year of life also influence these adaptations. At 3 months, infants exhibit significant sociability and a keen interest in face-to-face interactions (Tronick et al., 2020). At this age, maternal responses to infants in face-to-face interactions primarily involve facial expressions, responsive vocalizations, and touch (Stern et al., 1985). These social interactions impact infants' responsiveness and sociability (Bigelow and Rochat, 2006).

Later, around the second half of the 1st year of life, infants also begin to show interest in objects in addition to people. Joint attention, characterized by triadic exchanges where both the infant and a partner are aware of each other's shared focus on the same object or event, typically emerges between 6 and 9 months of age. These joint attention episodes mark infants' development of secondary intersubjectivity, indicating their awareness that experiences with objects can be shared with others (Shin, 2012). Can contextual shifts (e.g., attending daycare) and infant developmental acquisitions affect the continuity in parent-infant relationships? This topic is understudied, particularly in populations with infants at developmental risk like preterm. However, some found continuity in maternal sensitivity from 6 to 42 months (Pianta et al., 1989).

### Aims and hypotheses

Prematurity is a complex risk factor that includes social, family, parental, and infant health vulnerabilities. Infants born preterm, even those in the MLPT range, are at risk for lower cognitive, motor, and language development (e.g., Chung et al., 2020), health problems, brain injuries, and comorbidities compared to their FT counterparts (McCormick et al., 2011), which are associated with maternal trauma and parental stress (Fuertes et al., 2020b; Gonçalves et al., 2020).

The study is original in investigating: (1) the association between and (2) differences in maternal and infant interactive behavior from 3 to 9 months (continuity and discontinuity) in dyads with infants born FT, MLPT, and VEPT; and (3) comparing the mean differences in maternal sensitivity and infant cooperativity of the mothers and infants that maintained the same score (group SS) with the dyads that changed scored (group CS), at 3 and 9 months.

We expect interactions to evolve over these 6 months as partners become increasingly attuned and predictable regarding their interactive behavior. Therefore, we expect maternal sensitivity and infant cooperation to increase during this period. However, considering that prematurity affects developmental trajectories and family life, we expect a larger increase in dyads with infants born FT compared to the other two groups.

## Methods

### Participants

Participants were 213 Portuguese infants and their mothers, distributed by three independent samples: (1) the FT sample which included 105 infants born full-term (50 girls; 55 boys; M_gestationalage_ = 39.47; M_gestationalweight_ = 3,303), (2) the MLPT sample consisting of 52 infants born moderate-to-late preterm (22 girls; 30 boys; M_gestationalage_ = 33.84; M_gestationalweight_ = 2,108), and (3) the VEPT sample which included 56 infants born very-to-extreme preterm (26 girls; 30 boys; M_gestationalage_ = 29.45; M_gestationalweight_ = 1,155). Mothers of infants born VEPT were older, had fewer years of formal education, and had fewer children than mothers of FT infants. According to Hospital clinical records, no infants had any known sensory or motor impairment, severe illnesses, or congenital anomalies at delivery. Also, no parents had any history of mental health problems, and/or substance abuse.

### Procedures

#### Free-play interaction

When infants were 3 and 9 months (corrected age), mothers, recruited at birth, were recontacted to schedule a follow-up lab visit with their infants. At these visits, mother-infant dyads were videotaped in a free-play interaction. Following Crittenden Child-Adult Relationship Experimental Index (CARE-Index) instructions (Crittenden, 2003), each dyad was recorded for 5 min free. The CARE-Index includes three adult scales, namely Sensitivity, Control, and Unresponsiveness, and four infant scales, Cooperativity, Compliant-Compulsive, Difficulty, and Passivity. Each maternal and infant scale was scored regarding facial expressions, verbal expressions, position and body contact, affection, turn-taking, control contingencies, and choice of activity (from 0 to 14 points). Each scale (e.g., maternal sensitivity) includes descriptors for each dimension (e.g., facial expression). CARE-index is a dyadic measure, meaning that assessing each partner's interactive behavior implies considering the interactive context and its influence on the other partner. Infant and maternal behavior were scored by two reliable and blind (against the study hypotheses) coders. Intercoder reliability was evaluated by comparing the two coders' ratings using ICC-intraclass correlation coefficients (Cicchetti, 1994). The obtained overall average ICCs was 0.82.

## Analytic plan

Pearson correlations of maternal and infant interactive behavior at 3 and 9 months in all samples were used (aim 1). To address aims 2 and 3, descriptive analyses and a *t*-student test were used. Alpha was set at < 0.05, and the variables' normality was tested.

## Results

### First aim

According to Table 1, most maternal and infant behavior categories are correlated at 3 and 9 months, except for maternal unresponsivity in the FT sample, and infant difficulty, and passivity both in FT and MLPT samples.

**Table 1:** Means, standard deviations, and Pearson correlations of maternal and infant interactive behavior at 3 and 9 months in the three samples (FT, MLPT, and VEPT).

**Correlations at 3 and 9 months**	**FT**	**MLPT**	**VEPT**
**Maternal behavior**			
Sensitivity	0.531^***^	0.586^***^	0.323^*^
Control	0.294^**^	0.535^***^	0.473^***^
Unresponsivity	0.155	0.314^*^	0.464^***^
**Infant behavior**			
Cooperativity	0.370^***^	0.504^***^	0.390^**^
Compulsivity	0.227^*^	0.534^***^	0.530^**^
Difficulty	0.169	0.194	0.554^***^
Passivity	0.199	0.223	0.747^***^

^*^p < 0.05.

^**^p < 0.01.

^***^p < 0.001.

FT, infants born full-term; MLPT, infants born moderate-to-late preterm; VEPT, very-to-extreme preterm.

### Second aim

Table 2 shows no changes regarding maternal and infant interactive behavior in the VEPT sample from 3 to 9 months. In the MLPT group, only infant difficulty marginally increased and in the FT group, maternal unresponsivity, infant difficulty, and infant passivity increased while infant compulsivity decreased in the same period.

**Table 2:** Paired means differences test from maternal and infant interactive behavior from 3 to 9 months in the three samples (FT, MLPT, and VEPT).

		**FT**				**MLPT**				**VEPT**			
		***M***	***SD***	***t***	***p***	***M***	***SD***	***t***	***p***	***M***	***SD***	***t***	***p***
**Maternal behavior**													
Sensitivity	3 m	9.24	2.91	−1.918	0.06	8.15	2.508	0.533	0.60	7.46	2.26	1.837	0.07
	9 m	8.75	2.35			7.98	2.631			6.80	2.37		
Control	3 m	3.70	2.87	−1.027	0.31	3.81	3.17	−0.891	0.38	3.54	3.32	1.340	0.17
	9 m	3.38	2.54			4.17	2.95			4.20	3.82		
Unresponsivity	3 m	1.10	2.00	2.603	0.01	1.88	2.29	−0.052	0.96	3.00	3.27	−0.038	0.97
	9 m	1.78	2.09			1.90	2.30			2.98	3.42		
**Infant behavior**													
Cooperativity	3 m	8.87	2.43	1.488	0.14	7.77	2.63	−0.374	0.71	7.20	2.20	−0.428	0.67
	9 m	9.33	3.19			7.90	2.58			7.05	2.31		
Compulsivity	3 m	1.72	2.61	−2.059	0.04	2.60	3.48	−0.533	0.60	2.30	3.59	−0.354	0.72
	9 m	1.11	2.25			2.85	3.59			2.14	3.41		
Difficulty	3 m	1.05	2.21	−2.175	0.03	1.52	2.26	2.010	0.05	3.95	3.65	0.616	0.54
	9 m	1.65	2.18			2.42	2.80			4.23	3.70		
Passivity	3 m	1.86	2.33	−2.452	0.02	1.77	2.10	0.681	0.50	0.61	1.42	−1.090	0.28
	9 m	2.55	2.27			1.52	2.15			0.46	1.32		

FT, infants born full-term; MLPT, infants born moderate-to-late preterm; VEPT, very-to-extreme preterm.

### Third aim

Only 45 mothers obtained the same mean score on the maternal sensitivity scale at 3 and 9 months, distributed as follows: 16 mothers were from the VEPT sample (*N* = 56), 15 from the FT sample (*N* = 105), and 14 from the MLPT sample (*N* = 52). At 3 months, the mean of maternal sensitivity for mothers that maintained the same score (SS) from 3 to 9 months was at 6.89 (*SD*_*SS*_ = 2.10) compared to 8.93 for mothers that changed score (CS; *SD*_*Cs*_ = 2.75), *t*_(211)_ = 4.660; *p* < 0.001; *d* = 2.62. Also, the means of infant cooperative behavior at 3 months are significantly different in these two groups, M_SS_ = 6.77, *SD*_*SS*_ = 2.07 against M_Cs_ = 8.85, *SD*_*CS*_ = 2.99, *t*_(211)_ = 4.322; *p* < 0.001; *d* = 2.84). Similar results were obtained at 9 months. Therefore, mothers and infants who changed their scores had better interactive outcomes.

## Discussion

We aimed to study continuity and discontinuity in mother-infant interactions and found that discontinuity is associated with better interactive outcomes but is less likely in preterm infants.

First, we explored the association of maternal (sensitivity, control, and unresponsivity) interactive behavior at 3 and 9 months of age. Likewise, for infant interactive behavior (cooperativity, compulsivity, difficulty, and passivity). Maternal sensitivity, maternal control, infant cooperativity, and infant compulsivity were the interactive behaviors with higher association from 3 to 9 months in all samples. Supporting the Sameroff and Fiese (2000) transactional model, mothers and infants with the lower scores in these categories at 3 months also had lower rates at 9 months (and vice-versa).

Second, we tested the mean differences in maternal and infant interactive behavior from 3 to 9 months. No differences were found for dyads with infants born VEPT or MLPT from 3 to 9 months (and therefore, higher continuity). One possible explanation for these results is that mothers of infants born FT return earlier to their jobs, and probably their lives are more socially busy than mothers of infants born PT, challenging them to cope and adapt to those social changes. On the other hand, mothers of preterm infants may have more steady lives in the first 9 months of their infants' lives since they return later to their jobs (McCormick et al., 2011). Also, developmental acquisitions tend to be expressed earlier in FT infants (for instance, many infants can crawl or stand at 9 months) compared to PT infants. These emerging abilities indicate a shift in infants' exploration as they become more autonomous and oriented toward objects (Bigelow et al., 2004). These individual and contextual changes may be reflected in mother and infant interactions.

*Is discontinuity good?* Past research informs (Tronick, 2007) that interactions include moments of engagement, disengagement, and communication errors (cycles of interactive mismatches and repair). Yet, the ability to return to positive engagement and reciprocal interchanges from both partners, which requires flexibility and adaptation, characterizes reciprocal and positive relationships (Tronick et al., 2020). Corroborating this perspective, in our *third aim*, we found that the quality of maternal sensitivity and infant cooperative behavior is higher in dyads that change their score from the 3 to the 9-month visit compared to dyads that maintained the same score in both visits, regardless of the prematurity status. Thus, this study reinforces the importance of the capacity to cope and adapt to changes in relationships effectively. Early intervention programs aimed to enhance maternal sensitivity and infant cooperativity, can help promote reciprocal, positive, and developmentally adequate interactions (Dionisio et al., 2023), placing the child in a positive developmental trajectory.

Since in the VEPT sample, mothers were older and had fewer years of education; future studies should learn about the impact of these and other demographic factors on mother-infant interactive continuity, as found by past research (Granés et al., 2023).

Concerning the study limitations, the samples vary in sample size and risk status, which reduces the analysis's statistical power. However, our study is original in studying mothers and infants who maintained the same interactive profile from 3 to 9 months despite the critical infant developmental shifts that take place during this period.

This study also has several strengths. The use of direct observations and the inclusion of three independent samples (FT, MLPT, and VEPT). We believe the study's findings contribute to the growing knowledge about change and continuity in mother-infant interactions, particularly with infants born preterm.

## Data availability statement

The raw data supporting the conclusions of this article will be made available by the authors, without undue reservation.

## Ethics statement

The study was conducted in accordance with the Declaration of Helsinki, and approved by the Ethics Committee of Hospital de São João (protocol code CES 36-14 and date of approval 21-01-2015) for studies involving humans, and Conselho de Administração CHLO—Centro Hospitalar de Lisboa Ocidental (Entrada n° 2791, and date of approval 09-10-2015), as well as by the Portuguese Data Protection Commission. The studies were conducted in accordance with the local legislation and institutional requirements. Written informed consent for participation in this study was provided by the participants' legal guardians/next of kin. Written informed consent was obtained from the individual(s) for the publication of any potentially identifiable images or data included in this article.

## Author contributions

MF: Conceptualization, Data curation, Formal analysis, Funding acquisition, Project administration, Supervision, Writing—original draft, Writing—review & editing. JG: Data curation, Investigation, Validation, Visualization, Writing—review & editing.
